# Governing nonconventional genetic experimentation

**DOI:** 10.1093/jlb/lsad003

**Published:** 2023-03-09

**Authors:** Maxwell J Mehlman, Ronald A Conlon, Alex Pearlman

**Affiliations:** Case Western Reserve University, Cleveland, OH 44106, USA; Case Western Reserve University, Cleveland, OH 44106, USA; Concentric by Ginkgo, Boston, Massachusetts, USA

**Keywords:** Genetics, gene editing, nontraditional experimentation, regulation

## Abstract

A large and highly heterogeneous group of individuals conducts genetic and genomic research outside of traditional corporate and academic settings. They can be an important source of innovation, but their activities largely take place beyond the purview of existing regulatory systems for promoting safe and ethical practices. Historically the gene-targeting technology available for non-traditional genomic research has been limited, and therefore these activities have attracted little regulatory attention. New technologies such as CRISPR/Cas9, however, give nonconventional experimenters more extensive gene editing abilities at an unprecedented level of accessibility. The affordability and accessibility of these powerful technologies are raising questions about whether the current largely laissez-faire governance approach is adequate. This article recommends steps to enhance self-governance, including establishing umbrella organizations to represent community interests, creating a community IRB modelled on the DIYBio Ask a Safety Expert Service, and adopting an ethical obligation to report rogue experiments.

## I. BACKGROUND

We tend to think of sophisticated genetic experiments taking place in academic and corporate settings, but they also are conducted in community labs and people’s homes. Academic and corporate experimentation in the United States^1^ is highly regulated by the government and by the institutions in which it takes place. Until recently, there was little concern about harmful genetic experimentation outside of conventional settings because its capabilities were extremely limited,^2^ and regulators largely left it to govern itself. However, new technologies such as CRISPR/Cas9 give nonconventional experimenters more extensive gene editing abilities and are raising questions about whether the current largely laissez-faire governance approach is adequate. This article expands on a book chapter written by two of the authors.^3^ It begins by exploring the nature and history of nonconventional genetic experimentation (NGE) and its potential risks and benefits.^4^ After describing the current regulatory approach and other potential regulatory options, the authors argue that NGE should continue to be largely self-governed. The article concludes by suggesting steps to enhance self-governance that would not unduly restrict experimental freedom.

Nonconventional biology has a long and storied history. Indeed, some of the most notable advances in biology are attributed to nonconventional scientists. Charles Darwin has been called ‘the original do-it-yourself biologist’.^5^ Friar and later Abbot Gregor Mendel, recognized as the father of modern genetics, was the first to describe the laws of inheritance through his extensive experimentation on peas.^6^ Mary Anning was an amateur fossilist with numerous contributions to paleontology.^7^ Modern nonconventional biology has been described as a return to Victorian and earlier models of scientific endeavor.^8^

The members of the nonconventional biology community are highly diverse in terms of their background, interests, and values. Many nontraditional biologists rebel against the hierarchy and specialization of academic and corporate biology.^9^ Some are employed in traditional biological institutions but pursue projects of personal interest in their free time.^10^ Others are complete amateurs. As one commentator notes, ‘it is experimenting on the cheap, usually without the benefit of a fancy university laboratory …. If you don’t know enough biology to take part at first, you learn it along the way’.^11^ Nonconventional biology is pursued individually and in teams, in individual homes and group labs, and no doubt on a moonlight basis in industry and academic laboratories. In the spirit of the 1996 Bermuda Agreement^12^ and NIH policy on public access to genome sequencing data,^13^ many nonconventional experimenters reject intellectual property rights and insist on an open-source approach.^14^ Others actively seek to commercialize their discoveries. Some are public about their work, while others are secretive.^15^

Nonconventional biology is financed in a number of ways, including personal funding, dues to labs, investors, and crowdfunding, such as Kickstarter, which for example raised $35,000 to start a community laboratory called BioCurious in Silicon Valley^16^ and half a million dollars to create a company to sell bioluminescent plants.^17^ Individuals and labs use reverse engineering and inexpensive, off-the-shelf parts to construct simplified and low-budget equipment.^18^

As gene modification has become easier and more accessible with advances such as CRISPR (‘clustered regularly interspaced short palindromic repeats’), a technology used to alter DNA sequences,^19^ nonconventional biologists have begun to pursue gene editing.^20^ CRISPR/Cas9 is relatively accurate, inexpensive, and easy to use.^21^ Gene editing previously was practical only in a very limited number of cells and organisms, but CRISPR/Cas9 can be used to edit any cell or organism with a sequenced genome and with a way to introduce nucleic acids and/or proteins. As the former principal investigator of the Synthetic Biology Project at the Woodrow Wilson Center stated in 2016, ‘the equipment and reagents that are needed to use CRISPR-Cas9 are already readily available’. This is still true.^22^ Anyone can purchase a genome editing kit for as little as $200 and start tinkering with the delicate instructions of life.^23^ A recent report projected that the market for NGE supplies would reach over $52 million by 2027.^24^

The more basic NGE CRISPR experiments include creating a streptomycin-resistant strand of *Escherichia coli*,^25^ inserting a jellyfish gene into yeast to make glowing beer, and genetically modifying frogs to increase in size and weight.^26^ However, many amateurs are becoming increasingly sophisticated in their projects. Reagent introduction can be difficult for some target cells and organisms, but easier for others (eg direct injection into muscle, inhalation of aerosolized particles, microinjection of fish or amphibian eggs).^27^ Experimenters can select target organisms from the increasing number of organisms with sequenced genomes and obtain reagents and viral vectors from commercial suppliers, in some cases, as discussed below,^28^ by circumventing sales restrictions.^29^ One nontraditional biologist is described as ‘cutting, pasting and stirring genes, as simply as mixing a vodka tonic, … after which he slides his new hybrid creations, living in petri dishes, onto a refrigerator shelf next to the vegetables’.^30^ A dog breeder in the USA is using CRISPR technology to try to correct a genetic mutation that causes bladder stones in Dalmatians^31^ and an artist in the UK is using CRISPR to recreate the smell of extinct flowers, in collaboration with synthetic biology company Ginkgo Bioworks.^32^ NGE experiments involving self-injection of homemade genetic material already are underway to combat human disease^33^ and to enhance performance.^34^ Two recent approvals of gene therapies by the Food and Drug Administration (FDA) are likely to spark a resurgence of interest in improving gene delivery methods for therapeutic purposes, which could be used by NGE experimenters for human somatic gene editing.^35^ NIH Director Francis Collins states that human gene editing is ‘not the sort of thing you’d set up in your garage this afternoon. You still have to have a molecular biology lab with a certain skill set so it’s not trivial’. But he goes on to say that ‘compared to the ways that we’ve had previously available to do this, this is much more accessible and it’s much more inexpensive, maybe some people have said a thousand times cheaper than what we could’ve done before. So yes, there are concerns if it’s become that much more accessible, will there be a circumstance where somebody decides I don’t care what the ethicists say. I’m going to do this anyway and see if I can improve on a human being’.^36^ Harvard geneticist George Church estimates that ‘a person with molecular biology skills and know-how could probably do [human germ line gene editing] in a [sic] bio-hacker space for less than $2,000’, adding that most of what they would need can be built by NGE labs themselves.^37^

Nonconventional genetic experimentation (NGE) gained public attention in the early 2000s. An international competition for high school and college students called the ‘International Genetically Engineered Machine’ (iGEM) was founded in 2003 at MIT ‘to show that it does not take a doctoral degree to design a biotechnology product’.^38^ In a recent iGEM competition, 20 teams used CRISPR.^39^ An announcement in 2008 of the creation of a synthetic genome that did not exist naturally^40^ increased interest in genetic engineering, including NGE. A year later, Mac Cowell and Jason Bobe started ‘DIYBio.org’ as a shared space for NGE experimenters.^41^ Local, community, and regional labs were established, including BioCurious in Silicon Valley and GenSpace in New York.^42^ Equipment costs declined, and NGE experimenters reverse-engineered their own equipment.^43^ In 2015, an international effort called the Open Insulin Project debuted with the goal of using NGE to genetically engineer yeast and *E. coli* in order to produce insulin, which would be sold at a fraction of the cost of commercially manufactured products.^44^ In 2017, Josiah Zayner, who started a company called The Odin that sells NGE materials and equipment, including CRISPR kits, livestreamed injecting CRISPR-edited DNA in a vain effort to grow muscle by knocking out his myostatin gene.^45^ Also in 2017, the MIT Community Biotechnology Initiative held the first Global Community Bio Summit; the fifth was held in November 2021.^46^

## II. RISKS AND BENEFITS OF NGE

A major potential benefit of NGE, as with amateur science in the past, is innovation.^47^ In the case of computers, for example, Steve Jobs and Bill Gates did early work in their garages,^48^ and the first Apple was unveiled at a local electronics hobby club.^49^ NGE innovation can be fostered by experimenters having greater experimental freedom outside of traditional settings and more cross-disciplinary collaboration.^50^ NGE also can produce a broader pool of science talent^51^ and enhance science education.^52^

NGE also could be employed to improve access to certain types of health care. As mentioned earlier,^53^ the Open Insulin Project, which began in 2015 in a community lab in Oakland, aims to establish a network of community-based production sites to manufacture insulin and sell it to diabetics for $7 a vial^54^ rather than the current $175 to $300 cost for commercially manufactured products.^55^

As one set of commentators observes:

Biohacker spaces hold great potential for promoting innovation. Numerous innovative projects have emerged from these spaces. For example, biohackers have developed cheaper tools and equipment. They are also working to develop low-cost medicines for conditions such as diabetes …. On an economic level, biohacker spaces facilitate entrepreneurship by providing tools, training, and resources to help people prototype their biotechnology-based ideas. Biohacker spaces also help advance scientific research. There are many examples of ambitious projects that have derived from these spaces such as vegan cheese protein, genetically engineered bacteria that can sense arsenic, as well as robots that can automate lab work.^56^

The commentators note that NGE innovation can take place despite the conventional intellectual property protections that characterize traditional biomedical research:

Biohacker spaces demonstrate that innovation can arise outside of the formal IP system in a way that embraces open science and inclusivity …. A number of projects have emerged from biohacker spaces in the shadow of the formal IP system. For example, a group of biohackers developed Open Trons, an open source lab robot to automate lab work. The project originated from Genspace and raised well over $100,000 on Kickstarter, meeting 125% of its fundraising goal. Besides being a commercially successful campaign, the project also achieves other inclusive outcomes: it enhances understanding of lab automation through its open source technology and its low price compared to other lab automation robots enables access to this technology in labs that cannot afford the more expensive robots.^57^

Yet NGE creates safety risks. After 9/11, nonconventional biology raised concerns about bioterrorism, and the FBI placed its activities under surveillance.^58^ In 2004 and 2008, agents raided basement laboratories on suspicion of possession of dangerous chemicals.^59^ In 2006, the agency established the Weapons of Mass Destruction Directorate (WMDD), which continues to monitor nonconventional biological experimentation, and created within the Directorate the Synthetic Biology Outreach Program (SBOP) under Supervisory Special Agent Edward You.^60^ Since 2009, the FBI has sent observers to NGE meetings, conferences, and labs,^61^ including the infamous hacker convention, DefCon.^62^ Jason Bobe, co-founder of DIYBio, quipped in 2010 that ‘30% [of people on DIYBio e-mail] are spammers and the other 70% are law-enforcement officials keeping tabs on the community’.^63^

While its early response to nonconventional biology was aggressive, the FBI’s more recent approach is one of engagement and cooperation.^64^ In some cases, community labs keep agents apprised of their events and experiments.^65^ The FBI has organized annual NGE conferences where it fosters safety training, and the bureau has even sponsored iGEM, prompting some jokingly to observe that ‘DIYbio would not be where it is today without the FBI’s support’.^66^

A source of concern about NGE besides bioterrorism is the potential for experimenters to pose purposeful or accidental biosecurity threats by creating new or modified organisms that could harm human health or the environment. In an article in the *New Yorker*, for example, a science writer who admits having ‘almost no experience in genetics and [having] not done hands-on lab work since high school’ described how she was able to modify bacteria to make them drug-resistant using a ‘bacterial *CRISPR* and fluorescent yeast combo kit’ she purchased for $209 from Zayner’s California company, The Odin, which sells genetic engineering kits to NGE experimenters:

[B]y following the instructions that came in the box from the Odin, in the course of a weekend I was able to create a novel organism. First I grew a colony of *E. coli* in one of the petri dishes. Then I doused it with the various proteins and bits of designer DNA I’d stored in the freezer. The process swapped out one ‘letter’ of the bacteria’s genome, replacing an ‘A’ (adenine) with a ‘C’ (cytosine). Thanks to this emendation, my new and improved *E. coli* could, in effect, thumb its nose at streptomycin, a powerful antibiotic.^67^

Although in this particular case, streptomycin-resistant *E. coli* are prevalent and common in meat products in the United States due to agricultural use of streptomycin,^68^ modified bacteria could cause infections that were difficult or impossible to treat with available antibiotics. If released intentionally, such infectious agents could be a potent weapon of mass destruction.

Another worrisome possibility is that NGE experimenters could intentionally create and release modified organisms with ‘gene drives’, which enable genetic modifications to spread through wild populations much faster than normal and potentially replace natural genomes.^69^ Gene drives have been proposed, for example, as a means to combat the spread of malaria by mosquitoes and other insect-borne pathogens.^70^ An effective gene drive would introduce genetic modifications into the target insect population at a highly accelerated rate to eliminate or reduce the population or to modify the population so that it could not transmit the disease pathogen.^71^ Gene drives also could be used to combat agricultural pests and non-native species that threatened native plants and animals.^72^ But gene drives raise concerns about unintended consequences, such as off-target effects or the modified genes being transmitted to other closely related species.^73^

NGE also poses risks to subjects. These can be animals, humans, or the experimenters themselves, such as in the case of Josiah Zayner, as noted above. In another livestream in 2018, a biohacker injected himself with a treatment for genital herpes.^74^ If the subjects of NGE experiments were individuals other than the experimenter, questions might arise about whether they gave appropriate informed consent, and whether they were ill and seeking an experimental intervention hoping that it would be more effective or less expensive than conventional treatments^75^ In an NGE conference in Las Vegas in August 2019, for example, Zayner suggested that, to sidestep what he sees as accessibility and regulatory hurdles in conventional medicine, NGE experimenters who are collaborating with patients might consider working outside US borders in countries with less government oversight.^76^

Finally, despite its potential to increase accessibility to expensive therapeutics, NGE could have disruptive effects on the innovation models of traditional pharmaceutical and biotechnology industries. Under American patent law, an invention can be patented only if it is novel and nonobvious, which would be defeated if an NGE experimenter had created a sufficiently similar product or process and it was ‘described [it] in a printed publication, or in public use, on sale, or otherwise known to the public before’ the traditional biology innovator filed a patent application.^77^ As noted earlier, some NGE experimenters explicitly aim to thwart intellectual property rights and pursue an open-source approach to their discoveries, such as in the case of the biohackers who claimed to have made a copy of one of the world’s most expensive gene therapies, Glybera, in 2019.^78^

## III. THE CURRENT REGULATORY FRAMEWORK

### III.A. Law

In addition to the FBI, the FDA’s regulatory authority covers many aspects of NGE. To the extent that NGE experimenters are seeking to develop therapeutic interventions, such as Zayner’s myostatin treatment, the agency claims that they are producing and testing gene therapy products that come within the definition of ‘biologics’ in the Federal Food, Drug, and Cosmetic Act.^79^ In 2017, the FDA issued a statement entitled ‘Information About Self-Administration of Gene Therapy’ which asserted that it is illegal to sell ‘gene therapy products intended for self-administration and “do it yourself” kits to produce gene therapies for self-administration’ without an approved IND or Biologics License Application.^80^ Some commentators maintain that the FDA has broad jurisdiction over NGE because much of its materials and equipment are ‘drugs’.^81^

The ability of the FDA to regulate NGE, however, is substantially limited. For example, the agency lacks authority over interventions that experimenters make without using products or components in interstate commerce, as well as over instructions for experimentation that are not tied to a product or producer.^82^ Furthermore, the FDA’s broad authority to inspect commercial experimenters and manufacturers would not extend to inspecting NGE experimenters in their homes, which might require a warrant.^83^ Even if the agency wanted to regulate NGE, the resources needed to do so effectively would be prohibitive. As Evans states, ‘The staffing and budgets required to enforce regulations against thousands of tiny at-home operations could make regulation, as we knew it in the twentieth century, unworkable, especially if [NGE] grows more widespread than it is today’.^84^ Finally, while some NGE experimenters are eager to share their work with others on social media and at conferences, others are highly covert, and therefore may remain under the FDA’s radar.^85^ In the exercise of discretion, therefore, the FDA so far has not pursued any enforcement activities against specific NGE individuals or activities.^86^

The Environmental Protection Agency regulates microbial pesticides under the Federal Insecticide Fungicide and Rodenticide Act, as well as other recombinant DNA microbes and algae ‘intended for general commercial and environmental applications’ under the Toxic Substances Control Act,^87^ but ‘the reach of the law is limited to commercial or commercial research and development activities [and] it is unclear that ... noncommercial research efforts by DIY users are covered’.^88^ The Department of Agriculture’s Animal and Plant Health Inspection Service (APHIS) regulates field trials and commercial introduction only of plants genetically engineered through the use of known ‘plant pests,’ and has acknowledged that it lacks the authority to regulate crops modified with the latest gene editing techniques.^89^ In 1992 and again in 2017, the federal government revised the Coordinated Framework for the Regulation of Biotechnology Products,^90^ but the Framework gives federal agencies no new regulatory powers over biotechnology, and the revisions did not address drugs, biologics, or medical devices derived through biotechnology.^91^ NIH oversight is limited to research that is federally funded or conducted at academic institutions that receive federal funding. Intentional or reckless releases of engineered bioterror agents would be crimes,^92^ and humans whose health or property were harmed by such a release or by other types of unethical experimentation might seek to recover damages in civil suits^93^; but it may not be possible to hold the experimenters accountable because their identities may not be known, they may not be subject to US legal jurisdiction (for example, they may not be extraditable from a foreign country), or they may not have the assets to pay court-awarded damages.^94^

The only state law specifically addressing NGE was passed in California in 2019 as a response to Josiah Zayner’s 2017 public attempt to alter his myostatin gene.^95^ The bill, which was introduced by Republican state senator Ling Ling Chang and passed unanimously by the state legislature, prohibits the sale of ‘gene therapy kits’, defined as ‘a collection of materials for the purpose of facilitating gene therapy experiments’, unless the package bears a warning label stating that the kit ‘is not for self-administration’.^96^ According to an article in the *MIT Technology Review,* there are no products currently for sale that fit the definition of the kits in the law.^97^

### III.B. Non-Governmental Regulation

Some efforts at non-governmental regulation have been made by the biotech industry. In response to calls by the Department of Health and Human Services^98^ and the Department of Homeland Security^99^ for commercial suppliers of potentially dangerous reagents and synthetic genetic material to screen customers, members of the International Gene Synthesis Consortium and others voluntarily have agreed to ship reagents only to ‘valid business locations’, and to decline to ship to home addresses and post office boxes.^100^ However, NGE experimenters report that these suppliers do not verify whether their products are actually being shipped to a commercial establishment, as opposed to an experimenter’s personal address with a fictitious company name.^101^

The NGE community itself has taken a considerable number of self-regulatory steps. One of the reasons for the creation of DIYBio.org in 2009, according to co-founder Jason Bobe, was ‘for amateur biotechnologists to have a focal point … to think through issues that are beyond the scope of any one amateur, such as *safety and regulations for individuals working outside of traditional settings*’.^102^ In 2011, a North American DIYbio Congress adopted a code of ethics, described in 2013 as ‘to date perhaps the most explicit example of the community’s wish to find a “soft” yet binding way to create a common set of values and principles for its members’.^103^ A companion code was adopted in 2011 in Europe.^104^ However, the safety directive in both the North American and European codes merely states: ‘Adopt safe practices’.^105^

In 2013, DIYBio launched an ‘Ask a Biosafety Expert’ (ABE) service staffed by volunteers that responded to questions from nontraditional biologists.^106^ The service was discontinued due to the cost of liability insurance.^107^

In 2017, members of the NGE community convened the first Global Community Bio Summit, which has been followed by four others. The third meeting, held in 2019, adopted a ‘Community Ethics Document 1.0’ with 12 principles in the form of questions which organizers hoped could serve as an ethics ‘decision-making template’ for community labs and NGE researchers^108^ One, called ‘Transparency,’ asked: ‘How do we stay honest and open about our failures? How can we acknowledge ethical conflicts?’ Another principle, ‘Safety’, asked: ‘How do we embrace safe practices within unconventional contexts? How can we protect each other and create resources for communities to experiment safely?’ Finally, the principle of ‘Accountability’ asked ‘How will we hold each other accountable? How do we make ourselves accountable to people outside this community?’^109^

At the fourth Global Community Bio Summit in 2020, a group of community biosafety leaders launched a 260-page ‘Community Biology Biosafety Handbook’, described as ‘an open manual that offers biosafety protocols, practices, and recommendations aimed specifically for the community biology movement’.^110^ As an open manual, the format of the Handbook is a Google Doc:

The handbook is designed as a living document that can be updated and expanded by the community. Because both biotechnology and the Community Biology movement are rapidly evolving, the information here will need to be updated periodically. Also, given the variety of labs, the diverse local and national laws and regulations around labs, and the variety of interests among lab members, there will always be more that can be covered.^111^

The Handbook is focused on community labs. For example, it recommends that labs have ‘at least one point person who screens projects for safety (eg biological, physical, and chemical) and a committee of people to assist that point person in making decisions about safety and security’.^112^ The Handbook also recommends that labs ‘recruit expert advisors outside of the organization who you can consult when the safety of a project is in question or is beyond the expertise of the internal safety point person or committee. These consultants can be safety officers at universities, safety committees at other community biology labs, or colleagues in biotech or university labs who might be an expert in a field related to a proposed project’.^113^

Although the Handbook is focused on community labs, its preamble states that ‘it includes advice for … labs within homes and garages’.^114^ In addition to recommendations for how community labs can be run safely, it contains general information about mitigating safety risks associated with NGE, including risks to the environment and other organisms, including humans, from genetically engineering organisms.^115^ However, the Handbook currently does not provide specific safety recommendations for using newer gene editing techniques such as CRISPR, stating that ‘the biosafety requirements related to new methods of modifying the genome are still not entirely clear’, and that ‘no particular guidelines for working with these methods have been published by the NIH or other regulatory agencies’.^116^

The Biosafety Handbook also addresses the problem of desperate patients seeking cheaper or more effective treatments.^117^ After noting the emotional challenges and potential for legal risk and adverse publicity, the Handbook advises community labs to either ignore the inquiries, decline with compassion, or refer the inquirer to ‘professional resources’.^118^

Community labs promote safe experimentation in a number of other ways. Most of them restrict their members to Biosafety Level 1 experiments,^119^ which do not present significant health risks to humans or the environment.^120^ (However, at least one community lab, Counterculture Lab in Berkeley, California, allows Biosafety Level 2 experiments,^121^ which can pose moderate hazards.^122^) Community labs also typically require members to agree to adhere to safety codes of conduct. BioCurious, in Sunnyvale, California, for example, has the following Code of Conduct:

All protocols for workshops, and projects, or activities need to be run through the BioCurious Safety Group.

BioCurious must be informed of all reagents which will be entering the lab, ahead of time and the type of laboratory support required. This ensures safety for all.

Biosafety level 1 guidelines will be the limiting factor for projects, unless specifically noted otherwise. Only procedures which are approved and can be supported by BioCurious personnel and facilities will take place.

No human sampling or injecting of substances are allowed.

Genetically modified materials need to stay in the lab in which they were developed^123^.

Labs also usually require members to run their proposed experiments past a lab safety committee.^124^

During the height of the early months of the COVID-19 pandemic, a virtual community lab that hosted participants from around the globe, the Just One Giant Lab (JOGL) platform, became a hub for NGE activity related to open source work on coronavirus research, which raised the usual biosafety concerns. During this time, the JOGL community developed a biosafety advisory board and a unique set of biosafety guidelines as well as a Code of Conduct.^125^

Finally, the NGE community conducts robust conversations on social media platforms, such as the DIYbio.org Google Group, the Biohacking and Genetic Design Network, DIYBio, SyntechBio Biohacking Network, and Biohackers (GLOBAL), which are all on Facebook.^126^ These conversations include discussions about safety issues, such as inquiries and responses in 2020 about epigenetic gene therapy for rejuvenation^127^ and the safety of using lysed *E. coli* as an adjuvant in vaccine development.^128^

## IV. OTHER REGULATORY OPTIONS

If necessary, additional forms of regulation could be imposed on NGE. One option is greater governmental control. Certain other activities with robust independent research communities provide an analogy.

In the case of amateur or ‘sport’ rocketry, for example, the Federal Aviation Administration regulates the maximum height that sport rockets may reach.^129^ The Bureau of Alcohol, Tobacco and Firearms (BATF) requires permits for ‘toy’ rockets that are sold in interstate commerce, only grants permits to US citizens over the age of 18 who pass a background check, and limits propellants to 62.5 g. The US Department of Transportation regulates propellants as explosives under rules harmonized with the BATF. Legal violations carry both civil and criminal penalties.

In 2015, Congress passed a law governing both commercial and recreational drones and requires the two uses to be kept separate.^130^ With the passage of the National Defense Authorization Act of 2017, the FAA began requiring registration (for a $5 fee) if the weight of the recreational drone exceeds 0.55 pounds, prohibits weights over 55 pounds unless specially certified, and restricts operation near airports.^131^

Finally, federal law, enforced by the BATF, prohibits the sale of home brewing products and limits home production to 100 gallons per year per adult, with a maximum of 200 gallons per household, and some state laws restrict alcoholic content and shipment. Violations carry criminal penalties.^132^

In the case of NGE, the FBI could step up its monitoring of NGE experimentation, keeping a more wary eye out for questionable activities and even placing informants in NGE community labs and events. The FDA could increase regulation of NGE, and Congress could more clearly articulate the FDA’s authority to cover experimentation without commercial ambitions.^133^ At the extreme, states and the federal government could seek to make NGE illegal. German law, for example, prohibits gene editing outside of licensed laboratories.^134^ However, such draconian regulation might run afoul of Constitutional protection of free speech and scientific inquiry.^135^ It also would create incredible burdens for academic and industry researchers who might seek to collaborate with NGE projects and curtail the potential social and scientific advancements the community might offer.

Another option for NGE is indirect government regulation. For example, the government could make it illegal for suppliers to ship reagents to unapproved purchasers. As noted earlier, some suppliers voluntarily sell reagents only to ‘valid business locations’, but experimenters still get the reagents by using fictitious business addresses.^136^ In response, the government could require suppliers to verify more effectively the business address of the purchaser, inspect suppliers to ensure compliance, and penalize willful or negligent noncompliance.

An alternative to focusing reagent shipment restrictions on purchasers’ business addresses would be to require reagent purchasers to be licensed.^137^ Licenses could be issued by the government, or by an organization within the NGE community, such as a consortium of community labs. Precedent for the latter is found in the licensing scheme for amateur (‘ham’) radio, which is overseen by the Federal Communications Commission, but established and operated by the ham radio community itself through organizations such as the Amateur Radio Relay League (ARRL). Volunteers who are themselves licensed administer written ham radio licensing examinations, using questions prepared by volunteer-examiner coordinators in 13 regions that have coordinator agreements with the FCC.^138^

## V. JUDGING THE SUFFICIENCY OF THE CURRENT REGULATORY FRAMEWORK

Given the risks posed by NGE noted earlier, some commentators see a need for greater oversight. Landrain and colleagues, for example, state that ‘DIYbio still needs to find an active and, above all, *binding* way to deal with regulatory and safety issues’.^139^ Arthur Caplan and colleagues suggest creating ‘an international clearinghouse with which genetic sequence producers and sellers must register [and that] … would require all registered companies to monitor their orders and make sure that those who order biological material that could be misused have appropriate credentials, containment facilities, and training’.^140^ Wexler et al. highlight the importance of ‘community-developed standards and attention to sites of ethical gatekeeping’ as well as the imperative that the community ‘fosters partnerships between outside experts and DIY biologists’ to help develop community standards for biosafety.^141^ In 2010, the Presidential Commission for the Study of Bioethical Issues expressed the concern that ‘the DIY research communities and other private researchers are exercising such freedom but without the institutional norms and procedures designed to assure responsibility …’.^142^

Nevertheless, the current regulatory framework, with its focus on self-regulation, appears to be adequate at this time. Marketed results from NGE experiments seem to be effectively regulated by government agencies such as the FDA and the EPA. The FBI successfully monitors NGE activities for bioterrorism. Community labs seem to be doing a satisfactory job of overseeing experiments by their members, and there are no reports of dangerous lapses. Although there have been some troubling reports of self-experimentation outside of community labs, they do not appear to have produced serious harm, and if the self-experimenters are competent adults who are not engaging in interstate commerce, and are not violating any laws.^143^ Finally, work with infectious agents that have the potential for harm to humans, animals, plants, or the environment requires a level of sophistication not present in most home laboratory settings.

Furthermore, continuing to rely primarily on NBE self-governance yields important benefits. It affords experimental freedom, improving the prospect of socially and scientifically valuable discoveries. It allows for open and friendly collaboration with industry and academia, as the trend toward open science grows in popularity. It is consistent with the anti-establishment values of many NGE experimenters. Most importantly, it reduces the likelihood that experimenters would be driven underground, where they conducted risky experiments with less community awareness and potential oversight.

There is no way to be certain about the future, however. Overly ambitious experimenters could deliberately or inadvertently ignore community safety codes. Entrepreneurs could market more advanced equipment that enabled more elaborate experimentation. More powerful new gene editing techniques could emerge. It must be stressed that serious safety and ethical concerns would arise not only if NGE gene editing experiments were successful, but also if the apparent ease and relatively low cost of these techniques led to overly ambitious experiments that failed, causing harm to the environment and to human and nonhuman subjects.

Hence, the NGE community should consider forestalling greater external regulation by taking steps to enhance self-governance.

## VI. OPPORTUNITIES FOR ENHANCED SELF-GOVERNANCE

### VI.A. NGE Umbrella Organization

The NGE community could establish one or more umbrella organizations to accomplish certain community-wide tasks discussed below. One model is DIYBio. Founded in 2009, DIYBio created community-wide Facebook groups, blogs, and an ‘Ask a Biosafety Expert’ (ABE) Service, held meetings, and promulgated a Code of Ethics.^144^ Although the organization still has a website,^145^ it essentially ceased operations in 2018.^146^ Other potential successor organizations are the Global Community Bio Summit or JOGL, described above.^147^ Another possibility is for community labs to establish an umbrella organization.

One purpose for such an NGE organization would be to adopt ethical and safety recommendations for NGE experiments. As described above, the Global Community Bio Summit is pursuing this through its ongoing Community Biology Biosafety Handbook project.^148^ An additional step would be for the Bio Summit to invite individuals outside of the community, such as research, legal, and bioethics experts and government regulators, to review and comment on the recommendations, similar to what was done with the Human Augmentation Institute Draft Code of Ethics.^149^

Another function of community-wide organizations could be to represent the interests of the NGE community before the FDA and other federal agencies, Congress, and state legislatures and administrative agencies. Hobbies subject to external oversight such as ham radio, sport rocketry, drones, and home brewing all have such organizations. Ham radio operators are represented by the National Association for Amateur Radio, which goes by the initials ‘ARRL’ that stand for the Amateur Radio Relay League.^150^ Amateur or ‘sport rocketry’ enthusiasts have two groups, the National Association of Rocketry and the Tripoli Rocketry Association, which, in addition to promulgating safety codes^151^ and lobbying legislatures, bring lawsuits against overzealous government regulators and offer liability insurance to their members.^152^ These two organizations also belong to the National Fire Protection Association, a non-governmental organization that adopts model fire safety codes for rocket launches that are adopted in turn as is or in modified form by state and local governments.^153^ Recreational drone operators belong to a number of organizations including the Academy of Model Aeronautics and the Drone User Group.^154^ Home brewers, in an interesting twist, are represented by the American Home Brewers Association, a division of the commercial Brewers Association.^155^

### VI.B. NGE Community Review Board

Community labs, as noted earlier, typically have safety rules and individuals or committees to provide safety training and advice to members.^156^ Training and advice also is available at NGE meetings and competitions, such as iGEM’s Safety Hub.^157^ Since the demise of DIYBio’s Ask a Safety Expert Service, however, no consultative resources are available for independent experimenters working on their own^158^ outside the JOGL platform, which still offers a place for members to ask questions on Slack. Therefore, one important self-regulatory step would be to re-establish such a resource.

One option is ‘community review,’ whereby experimenters would present their proposed projects to the NGE community at large, such as via a blogpost, and receive comments from whatever members who chose to respond.^159^ This could be a valuable resource, but its usefulness would depend on which members participated and how expert they were in the issues presented. Moreover, projects posted on the web are likely to either be completely public or widely viewed by members of the community, which could deter experimenters who preferred to keep their projects confidential.

A more formally organized community review board (CRB) modeled on the DIYBio’s Ask a Safety Expert Service would be an additional valuable resource. It would resemble the institutional review boards (IRBs) that are required by law to review and approve human experiments that are funded by the federal government or that research biomedical products needing government approval before they can be marketed. Submission of projects for review by the CRB, however, would be voluntary.

The idea of an IRB-type entity for ‘citizen scientists’ appears to have been broached first in a Scientific American Blog by Jessica Richman and Zachary Apte in 2013.^160^ Calling it a ‘mini-IRB,’ they described it as:

A publicly available IRB for citizen scientists, which would ensure that the creators had undergone ethics training, had considered how their project would affect the participants, that was available at a fairly low (and fixed) cost and could be achieved within a finite amount of time. This ‘mini-IRB’ would not be a full review, but would ensure that it was clear who was responsible for the project, that they had done their due diligence, and that participants were informed of the risks and benefits.^161^

The third Global Community Bio Summit discussed creating what they called a ‘Biohacker IRB’.^162^ Members of JOGL have also indicated interest in establishing an NGE IRB.^163^

Establishing a CRB faces several challenges. First, a process would be needed to identify the expert members. Like the experts who served on the Ask a Safety Expert Service, they would be volunteers. They could be selected by the community organization described earlier or by individual or groups of community labs, or they could be nominated or self-nominated. They could be encouraged to be Registered Biosafety Professionals and Certified Biological Safety Professionals with the American Biological Safety Association like the members of the Ask a Safety Expert Service,^164^ or these or similar credentials could be required. To promote community acceptance, members of the community could be invited to vote on the candidates. They could serve without compensation, or they could be given honoraria. Either way, like the members of the Ask a Safety Expert Service, they should be covered by CRB liability insurance in case someone was injured in an experiment that they had reviewed.^165^

Operating a CRB requires funding. As noted earlier, DIYBio’s Ask a Safety Expert Service was discontinued due to the cost of liability insurance. CRB members might be given honoraria, and the CRB needs administrative support to manage its operations. The NGE community should consider the recommendation made by Jessica Richman and Zachary Apte in their 2013 blog mentioned above that funds be obtained from the NIH or the National Science Foundation or from crowdfunding platforms such as Microryza or Medstartr.^166^

As stated above, consultations with the CAB would be voluntary. Voluntary consultation could be incentivized, however. The CRB could arrange for liability insurance for experimenters who submitted their projects for CRB review; similar insurance is offered to members of the National Association of Rocketry,^167^ the Academy of Model Aeronautics,^168^ and the Home Brewers Association.^169^ In addition, NGE experimenters who followed the advice of the CRB could assert that as a defense in lawsuits arising from harm caused by their experiments. Ideally, courts would regard a favorable CRB review or compliance with CRB recommendations as creating a rebuttable presumption of reasonableness. A complainant could rebut the presumption by showing through eyewitness testimony or physical evidence that the experimenter failed to follow the protocol that the CRB favorably reviewed or to incorporate modifications to the protocol recommended by the CRB. Experimenters would continue to be liable for harm caused intentionally or recklessly. Finally, experimenters could request that projects submitted to the CRB be kept confidential.

Institutional Biosafety Committees (IBCs) and Biosafety Officers are additional safety measures at traditional research institutions. As noted earlier, many community labs have similar resources, and all labs should follow suit. Since IBCs and Biosafety Officers at traditional institutions primarily ensure that the institutions comply with regulatory requirements to minimize liability,^170^ they do not seem critical for independent NGE experiments so long as the experimenters have access to a suitable CRB.

### VI.C. Community Policing

Currently, experiments that raise safety or ethical concerns may elicit community opprobrium on community social media platforms mentioned earlier.^171^ Additional steps might be for community labs and other NGE organizations to designate individuals to monitor blogs, social media, and newspaper and journal accounts, and for the community to make it clear that anyone who is aware of a questionable experiment is expected to get the word out.

The community’s formal means of enforcing its standards currently are limited to forbidding participation in community activities such as labs and conferences. The Community Biology Biosafety Handbook, for example, states:

Removing members is often a very difficult but sometimes a necessary part of running a community organization. It is important to set standards for conduct and also demonstrate to the community that leadership is willing to hold members to account for their conduct inside as well as outside of the lab. It is important to set up and understand the process of removing and banning a member. These procedures should be documented in your Code of Conduct, Membership Agreement, and possibly other places like your waiver and bylaws depending on your legal structure. Removal could include immediate dismissal during a class, imposing a temporary cool-off period, or a permanent ban from the community lab. These policies should be documented in your code of conduct and adhered to.^172^

The Handbook also recommends that these cases be documented ‘from many points of view in an incident report form’, noting that ‘these documents may be legally required (eg by corporate structure) or be involved in legal proceedings’ and that ‘lab leadership should seek to resolve these issues thoroughly and quickly’.^173^

Community disapproval and banishment from community activities may not adequately deter rogue experimenters, however. The limitations on self-regulatory sanctions may explain why no similar amateur activities such as drones, computer hacking, amateur rocketry, and ham radio, rely entirely on self-regulation.

An additional deterrent would be to report serious safety concerns to external authorities, such as the FBI’s Directorate the Synthetic Biology Outreach Program (SBOP).^174^ A reporting process could be formalized, for example, in community lab policies, codes of conduct, and membership agreements.

A greater concern is problematic experiments performed independently outside of community labs and existing networks, where members are familiar with each other. The Community Biology Biosafety Handbook currently is silent on whether members of the community who become aware of rogue experiments are expected to report them to regulators, and although the Handbook recommends that community labs document troubling cases for use in legal proceedings,^175^ members of the community who merely fail to report a harmful experiment that they become aware of, as opposed to an experiment that they actively facilitate or conceal, are unlikely to be successfully prosecuted or sued.^176^ So reporting seemingly is a matter of individual conscience. As a means of forestalling additional external regulation, the NGE community could adopt an ethical obligation to report, such as incorporating it in the Biosafety Handbook.

## VII. CONCLUSION

Nontraditional genetic experiments can make important contributions to science, medicine, and society. The best way to balance the risks and benefits is to accord the experimenters a considerable degree of self-governance. By enhancing its tools of self-governance, the NGE community can forestall increased external regulation and maximize its experimental freedom.

It must be stressed, however, that given the potential risks from inappropriate NGE, the NGE community and society generally must continue to monitor NGE experimentation carefully to ensure that community self-governance effectively regulates experimental activities. Community-wide gatherings such as the Global Community Bio Summits should receive regular community monitoring updates so that additional self-governance measures can be adopted as needed. If serious safety lapses occur, additional forms of external regulation discussed earlier may be necessary.

## FUNDING

Research funding for this article was provided by the National Human Genome Research Institute, National Institutes of Health (1R03HG010256-01A1).

## Footnotes

1 This article focuses on nonconventional genetic experimentation in the United States. It discusses developments in other countries where relevant to the United States.

2 See Katrine S. Bosley et al., *CRISPR Germline Engineering—The Community Speaks*, 33 Nat. Biotechnol. 478–86 (2015); Thomas Landrain et al., *Do-It-Yourself Biology: Challenges and Promises for an Open Science and Technology Movement*, 7 Syst. Synth. Biol. 115–26 (2013); Presidential Commission for the Study of Bioethical Issues, New Directions: The Ethics of Synthetic Biology and Emerging Technologies 12 (2010) [hereinafter Presidential Commission].

3 Maxwell Mehlman & Ronald Conlon, *Governing Nontraditional Gene Editing*, in Consumer Genetic Technologies: Ethical and Legal Considerations 145–56 (Cohen, G., Farahany, N., Greely, H., and Shachar, C. eds., Cambridge University Press 2021).

4 Some refer to these experiments as ‘do-it-yourself biology’ (Landrain et al., *supra* note 2, at 115), but many experiments are carried out in community labs or with the help of friends and virtual groups. Some call the experiments ‘bio-hacking,’ (Heidi Ledford, *Garage Biotech: Life Hackers*, 467 Nature 650–2 (2010); Günter Seyfried, Lei Pei & Markus Schmidt, *European Do-It-Yourself (DIY) Biology: Beyond the Hope, Hype and Horror*, 36 Bioessays 548–51 (2014)), but ‘hacking’ has negative connotations for some that could belie the potential societal benefits from these efforts, discussed *infra* at pp. 8–12. Others refer to these experiments as ‘citizen science’ (Julian Guthrie, *Do-It-Yourself Biology Grows with Technology*, SFGATE (Dec. 20, 2009), https://www.sfgate.com/science/article/Do-it-yourself-biology-grows-with-technology-3277834.php), but that includes more passive public participation in projects such as NIH’s ‘All of Us’ program. The authors therefore prefer the term ‘NGE’.

5 Guthrie, *supra* note 4.

6 Mauricio De Castro, *Johann Gregor Mendel: Paragon of Experimental Science*, 4 Mol. Genet. Genomic Med. 3–8 (2016).

7 Rachel Kaufman published, *Mary Anning: Life and Discoveries of the First Female Paleontologist*, LiveScience (Feb. 22, 2021), https://www.livescience.com/who-was-mary-anning.html.

8 Prashant Nair, *Straight Talk with...Mac Cowell and Jason Bobe*, 15 Nat. Med. 230–1 (2009).

9 See Landrain et al., *supra* note 2, at 117; Hanno Charisius, Richard Friebe & Sascha Karberg, *Becoming Biohackers: Learning the Game*, BBC (Jan. 23, 2013), https://www.bbc.com/future/article/20130122-how-we-became-biohackers-part-1; and Seyfried et al., *supra* note 4, at 551.

10 Victoria Stodden, *Open Science: Policy Implications for the Evolving Phenomenon of User-Led Scientific Innovation*, 9 Jcom A05 (2010).

11 Spencer Michels, *What is Biohacking and Why Should We Care?*, PBS NewsHour (Sept. 23, 2014), https://www.pbs.org/newshour/science/biohacking-care.

12 Alessandro Delfanti, *Biohackers: The Politics of Open Science,* London: Pluto Press; 2013, at 158; Bermuda Agreement: Wellcome Trust; 1996 [updated Jan. 28, 2016].

13 NHGRI. Policy on Availability and Patenting of Human Genomic DNA Sequence Produced by NHGRI Pilot Projects 1996 [updated Jan. 28, 2016].

14 Delfanti, *supra* note 12; Ledford et al, *supra* note 4, at 651; Seyfried et al., *supra* note 4, at 548, 551; NHGRI, *supra* note 13; *Biohackers of the World, Unite*, The Economist (Sept. 4, 2014), https://www.economist.com/technology-quarterly/2014/09/04/biohackers-of-the-world-unite; Andrew Fazekas, *DIY Biology: How* Amateur *Scientists Are Playing with Genetic Code*, Yahoo News (Sept. 16, 2015), https://ca.news.yahoo.com/blogs/geekquinox/diy-biology--how-amateur-scientists-are-playing-with-genetic-code-191755522.html.

15 Ledford, *supra* note 4, at 650.

16 Landrain et al., *supra* note 2; Ledford, *supra* note 3, at 652; Fazekas, *supra* note 14.

17 Fazekas, *supra* note 14, at ¶ 11.

18 Landrain et al., *supra* note 2, at 650; James Bloom, *The Geneticist in the Garage*, The Guardian (Mar. 19, 2009), https://www.theguardian.com/technology/2009/mar/19/biohacking-genetics-research.

19 Elizabeth Kolbert, *CRISPR and the Splice to Survive*, New Yorker (Jan. 11, 2021), https://www.newyorker.com/magazine/2021/01/18/crispr-and-the-splice-to-survive.

20 Kristen V. Brown, Inside the garage labs of DIY gene hackers, whose hobby may terrify you, Project Earth (Mar. 29, 2016); Ellen Jorgensen, *Opinion | How DIY Bio-Hackers are Changing the Conversation Around Genetic Engineering*, Washington Post (May 20, 2016), https://www.washingtonpost.com/news/in-theory/wp/2016/05/20/how-diy-bio-hackers-are-changing-the-conversation-around-genetic-engineering; Emily Baumgaertner, *As D.I.Y. Gene Editing Gains Popularity, ‘Someone Is Going to Get Hurt’*, New York Times (May 14, 2018), https://www.nytimes.com/2018/05/14/science/biohackers-gene-editing-virus.html.

21 Bosley et al., *supra* note 2; Maxmen A, *Easy DNA Editing Will Remake the World. Buckle Up*, Wired (2015), http://www.wired.com/2015/07/crispr-dna-editing-2/;*Next-Generation Genome Editing*, 33 Nat. Biotechnol. 429 (2015).

22 Todd Kuiken, *Governance: Learn from DIY biologists*, 531 Nature 167–8 (2016).

23
*Id.*

24
*Insights on the Biohacking Global Market to 2027 - by Type, Product, Application, End-Use and Regions*, PRNewswire (Mar. 4, 2021), https://www.prnewswire.com/news-releases/insights-on-the-biohacking-global-market-to-2027---by-type-product-application-end-use-and-regions-301240909.html.

25
*Id.*

26
The Odin, https://www.the-odin.com/all-products/ (last visited July 25, 2022).

27 See Hao Yin, Kevin J. Kauffman & Daniel G. Anderson, *Delivery Technologies for Genome Editing*, 16 Nat. Rev. Drug Discov. 387–99 (2017).

28 See *infra* p. 14.

29 BioMaster, Comment to *Does NEB Send to Private Individuals?*, Google Groups (Aug. 18, 2016), https://groups.google.com/g/diybio; Emily Mullin, *A Biotech CEO Explains Why He Injected Himself with a DIY Herpes Treatment on Facebook Live*, MIT Technol. Rev. (2018), https://www.technologyreview.com/2018/02/05/145817/a-biotech-ceo-explains-why-he-injected-himself-with-a-diy-herpes-treatment-live-on-stage/.

30 Lisa Krieger, *Bay Area Biologist’s Gene-Editing Kit Lets Do-It-Yourselfers Play God at the Kitchen Table*, The Mercury News (Jan. 11, 2016), https://www.mercurynews.com/2016/01/11/bay-area-biologists-gene-editing-kit-lets-do-it-yourselfers-play-god-at-the-kitchen-table/.

31 Andrew Rosenblum, *A Biohacker’s Plan to Upgrade Dalmatians Ends Up in the Doghouse*, MIT Technology Review, Feb. 1. 2017, https://www.technologyreview.com/2017/02/01/243683/a-biohackers-plan-to-upgrade-dalmatians-ends-up-in-the-doghouse/.

32 Sigal Samual, *How Biohackers are Trying to Upgrade Their Brains, Their Bodies – and Human Nature*, Vox (Nov. 15, 2019), https://www.vox.com/future-perfect/2019/6/25/18682583/biohacking-transhumanism-human-augmentation-genetic-engineering-crispr; Alexandra Daisy Ginsberg, *Resurrecting the Sublime 2019*, https://www.daisyginsberg.com/work/resurrecting-the-sublime. An article in the *New Yorker* provided this description of one NGE experimenter who focuses on flowers: ‘In early March, I spent an afternoon with Sebastian Cocioba, a twenty-nine-year-old self-taught plant biologist who has built a remarkable lab in his parents’ Long Island City apartment by making clever use of items purchased on eBay and what he calls “a little bit of electronics know-how.” In a small spare bedroom, Cocioba has a PCR machine (“one of the schmanciest, with an actual freakin touch screen!”); a gene gun, for injecting DNA or RNA into cells; a laminar-flow hood; a centrifuge; a vortex shaker, for mixing fluids; and shelves lined with bottles of chemicals and proteins. He put together this suite of equipment at a cost of about seven thousand dollars. (For fun, he’s affixed googly eyes to some of the machines.) Cocioba works on plant-tissue cultures and genetic engineering, and has expertise in designing and producing flowers with new colors and patterns. In his parents’ kitchen, the white cabinets are covered in notes, scrawled in Sharpie, from a meeting that Cocioba had several months ago with a Japanese businessman and an N.Y.U. biologist, who were hoping to create a specially patterned morning glory as a symbol for the Tokyo 2020 Olympics. Cocioba’s mom, who sometimes doubles as his lab manager, likes how the marked-up cabinets look. On the refrigerator was a list describing the steps involved in engineering a blue rose’. Margaret Talbot, *The Rogue Experimenters*, New Yorker (May 18, 2020), https://www.newyorker.com/magazine/2020/05/25/the-rogue-experimenters.

33 Brown, *supra* note 20; Rowan Jacobsen, *Hacking Your Genes Has Never Been Easier*, Outside Online (Sept. 6, 2017), https://www.outsideonline.com/outdoor-adventure/environment/ultimate-life-hack/.

34 Patricia J. Zettler, Christi J. Guerrini & Jacob S. Sherkow, *Regulating Genetic Biohacking*, 365 Science 34–6 (2019); Adele Peters, *Modifying Your Own Genes Is Just An Injection Away–If You’re Feeling Lucky*, Fast Company (Oct. 10, 2017), https://www.fastcompany.com/40477808/genetic-modification-is-just-an-injection-away-if-youre-feeling-lucky; Brian Wang, *CRISPR Muscle Boosting Gene Therapy at Few Thousand Dollars Per Year Will Go Mainstream,*NextBigFuture (Nov. 18, 2017), https://www.nextbigfuture.com/2017/11/diy-biohacking-with-crispr-gene-therapy-for-muscle-boosting-myostatin-inhibitor.html.

35 Press Release, U.S. Food and Drug Administration, *FDA Approval Brings First Gene Therapy to the United States* (Aug. 30, 2017), https://www.fda.gov/news-events/press-announcements/fda-approval-brings-first-gene-therapy-united-states; Press Release, U.S. Food and Drug Administration, *FDA Approves CAR-T Cell Therapy to Treat Adults with Certain Types of Large B-Cell Lymphoma* (Oct. 18, 2017), https://www.fda.gov/news-events/press-announcements/fda-approves-car-t-cell-therapy-treat-adults-certain-types-large-b-cell-lymphoma.

36
*Latest Advances in Gene Editing: The Possibilities and Risks*, The Diane Rehm Show (Jan. 5, 2016), http://thedianerehmshow.org/shows/2016-01-05/gene-editing-the-possibilities-and-risks.

37 Kevin Loria, *The Process Used to Edit the Genes of Human Embryos Is So Easy You Could Do It in a Community Bio-Hacker Space*, Business Insider (May 11, 2015), https://www.businessinsider.com/how-to-genetically-modify-human-embryos-2015-4.

38 Erik Parens, Josephine Johnston & Jacob Moses, *Ethical Issues in Synthetic Biology: An Overview of the Debates* at 15 (Woodrow Wilson International Center for Scholars, June 2009), https://www.wilsoncenter.org/sites/default/files/media/documents/publication/synbio3.pdf.

39 Loria, *supra* note 37.

40 Daniel G. Gibson et al., *Complete Chemical Synthesis, Assembly, and Cloning of a Mycoplasma Genitalium Genome*, 319 Science 1215–20 (2008).

41 Delfanti, *supra* note 12; Landrain et al., *supra* note 2; Ledford, *supra* note 4; Seyfried et al., *supra* note 4; Presidential Commission, *supra* note 2.

42 Delfanti, *supra* note 12; Landrain et al., *supra* note 2; Charisius et al., *supra* note 9.

43 Landrain et al., *supra* note 2; Bloom, *supra* note 18.

44 Talbot, *supra* note 32, at ¶ 2.

45
*Id.*

46 See Global Community Bio Summit, https://www.biosummit.org/2021 (last visited July 25, 2022).

47 Rob Carlson, *Splice It Yourself*, Wired (May 1, 2005, 12:00 PM), https://www.wired.com/2005/05/splice-it-yourself/; Ledford, *supra* note 4, at 652; Fazekas, *supra* note 14, at ¶ 3; Gaymon Bennett et al., *From Synthetic Biology to Biohacking: Are We Prepared?*, 27 Nat Biotechnol 1109–11 (2009). A 2009 report of the National Security Council noted that ‘the beneficial nature of life science research is reflected in the widespread manner in which it occurs. From cutting-edge academic institutes, to industrial research centers, to private laboratories in basements and garages, progress is increasingly driven by innovation and open access to the insights and materials needed to advance individual initiatives’. National Security Council, Exec. Office of the President, National Strategy for Countering Biological Threats (2009), https://obamawhitehouse.archives.gov/sites/default/files/National_Strategy_for_Countering_BioThreats.pdf.

48
*Steve Jobs and Steve Wozniak*, Lemelson, https://lemelson.mit.edu/resources/steve-jobs-and-steve-wozniak (last visited July 25, 2022).

49
*‘First Apple Computer’ Sells for $815,000*, BBC News (Aug. 26, 2016), https://www.bbc.com/news/technology-37199000.

50 Nair, *supra* note 8.

51 Ledford, *supra* note 4, at 650.

52 Francis S. Collins, *Statement on NIH Funding of Research Using Gene-Editing Technologies in Human Embryos*, Nat’l Institute of Health (Apr. 28, 2015), http://www.nih.gov/about-nih/who-we-are/nih-director/statements/statement-nih-funding-research-using-gene-editing-technologies-human-embryos.

53 See *supra* text accompanying note 44*.*

54 Talbot, *supra* note 32, at ¶ 2.

55 Joshua Cohen, *Insulin’s Out-Of-Pocket Cost Burden to Diabetic Patients Continues to Rise Despite Reduced Net Costs to PBMs*, Forbes (Jan. 5, 2021), https://www.forbes.com/sites/joshuacohen/2021/01/05/insulins-out-of-pocket-cost-burden-to-diabetic-patients-continues-to-rise-despite-reduced-net-costs-to-pbms/.

56 Jeremy de Beer & Vipal Jain, *Inclusive Innovation in Biohacker Spaces: The Role of Systems and Networks.* 8 Technol. Innov. Mgmt. Rev. 27–37 (2028) (citations omitted). http://doi.org/10.22215/timreview/1137.

57
*Id.* (citations omitted).

58 Bloom, *supra* note 18; Bennett et al., *supra* note 47; Arthur L. Caplan et al., *No Time to Waste—The Ethical Challenges Created by CRISPR*, 16 EMBO Rep. 1421–6 (2015); Michele S. Garfinkel et al., *Synthetic Genomics | Options for Governance*, 5 Biosecur. Bioterror. 359–62 (2007).

59 Landrain et al., *supra* note 2; *Hacking Goes Squishy*, The Economist: Technology Quarterly (Sept. 2009), https://www.economist.com/technology-quarterly/2009/09/05/hacking-goes-squishy (In 2004, the FBI detained an art professor after his wife died and they found biological specimens in a basement laboratory.); *Charge Dropped Against Artist in Terror Case*, New York Times (Apr. 22, 2008), https://www.nytimes.com/2008/04/22/nyregion/22bioart.html (Although no hazardous materials were found, he and another individual were indicted for mail fraud for transporting the materials in violation of a university Materials Transfer Agreement, although the indictment later was quashed.); Bloom, *supra* note 19 (In 2008, state environmental officials and the FBI tore apart another basement laboratory belonging to a retired chemist on suspicion that he was working with dangerous chemicals.).

60
*Charge Dropped Against Artist in Terror Case, supra* note 59.

61 Ledford, *supra* note 4.

62
https://www.youtube.com/watch?v=qDEJMc9JiJ0.

63 Ledford, *supra* note 4.

64 Presidential Commission, *supra* note 2; Sara Tocchetti & Sara Angeli Aguiton, *Is an FBI Agent a DIY Biologist Like Any Other? A Cultural Analysis of a Biosecurity Risk*, 40 Sci. Technol. & Hum. Values 825–53 (2015); Heidi Ledford, *Biohackers Gear Up for Genome Editing*, 524 Nature 398–9 (2015).

65 Guthrie, *supra* note 4.

66
*Biohackers of the World, Unite*, *supra* note 14.

67 Kolbert, *supra* note 19, at ¶ 8.

68 Shaohua Zhao, Karen Blickenstaff, Sonia Bodeis-Jones, Sarah A. Gaines, Emily Tong, and Patrick F. McDermott, *Comparison of the Prevalences and Antimicrobial Resistances of Escherichia coli Isolates from Different Retail Meats in the United States, 2002 to 2008*. 78 Appl. Environ. Microbiol. 1701–7 (2012).

69 ‘A gene drive consists of three key components: the gene that you want to spread; the Cas9 enzyme that can cut DNA; and CRISPR, a pro[gra]mmable DNA sequence that identifies where the enzyme should cut. The genetic material that encodes for those three elements gets inserted into an animal’s DNA, in place of the naturally occurring gene you want to replace in both chromosomes. The power of the gene drive is that it disrupts the laws of heredity…. In normal heredity, there is a 50% chance that any particular gene will be passed from parent to offspring. Gene drive technology turns a 50% chance into a nearly 100% guarantee. When an animal carrying the gene drive package mates with an animal that does not, their offspring gets one copy of DNA from either parent: a natural version and a gene drive version. When the sperm meets the egg and the chromosomes from the different parents line up for the first time, CRISPR in the gene drive DNA is activated. It recognizes the copy of the natural gene in the opposite chromosome, and directs the DNA-cutting Cas9 enzyme to cut out the natural copy before embryonic development begins. Once the natural gene is damaged, the cell’s special repair machinery is triggered. The repair machinery restores the missing DNA, but it uses the unbroken chromosome, which is the one carrying the gene drive, as its template. So when the repair is finished, both chromosomes carry a copy of the gene drive. From that point on, two copies of the gene drive will be in every cell and the animal will pass the gene drive on to the next generation. And so the process continues. Every time the drive is passed on, CRISPR cuts the natural version of the gene, cell repair machinery intervenes and one copy of the gene drive becomes two. In just a few generations the new gene becomes ubiquitous in the population, sometimes totally replacing the naturally occurring gene.’ Donavyn Coffey, *What Is a Gene Drive?*, LiveScience (Apr. 17, 2020), https://www.livescience.com/gene-drive.html.

70 Ethan Bier, *Gene Drives Gaining Speed*, 23 Nat. Rev. Genet. 5–22 (2022).

71
*Id*. at 7.

72
*Gene Drives Promise Great Gains and Great Dangers*, The Economist (Nov. 10, 2018), https://www.economist.com/leaders/2018/11/08/gene-drives-promise-great-gains-and-great-dangers.

73
*Id*.; Jackson Champer, Anna Buchman & Omar S. Akbari, *Cheating Evolution: Engineering Gene Drives to Manipulate the Fate of Wild Populations*, 17 Nat. Rev. Genet. 146–59 (2016).

74
*Id*. Talbot notes that ‘[i]t’s unclear if it worked; Traywick died later that year, in Washington, D.C., while using a sensory-deprivation tank’.

75 Zettler et al., *supra* note 34.

76 Talbot, *supra* note 32.

77 35 U.S. Code § 102(a)(1).

78 Alex Pearlman, *Biohackers Are Pirating a Cheap Version of a Million-Dollar Gene Therapy*, MIT Technology Review, Aug. 30, 2019, https://www.technologyreview.com/2019/08/30/133193/biohackers-are-pirating-a-cheap-version-of-a-million-dollar-gene-therapy/.

79
*Information About Self-Administration of Gene Therapy*, U.S. Food and Drug Administration (Nov. 11, 2017), https://www.fda.gov/vaccines-blood-biologics/cellular-gene-therapy-products/information-about-self-administration-gene-therapy.

80
*Id*.

81 See Zettler et al., *supra* note 34.

82 Christi J. Guerrini, G. E. Spencer & Patricia J. Zettler, *DIY CRISPR*, 97 N.C. L. Rev. 1399, 1434 (2019), https://scholarship.law.unc.edu/nclr/vol97/iss5/17.

83 Barbara J. Evans, *Programming Our Genomes, Programming Ourselves*, in Consumer Genetic Technologies (Cohen et al., eds. Cambridge University Press 2021).

84
*Id.*

85 Zettler et al., *supra* note 34.

86
*Id*.

87 Executive Office of the President Office of Science and Technology Policy, Coordinated Framework for Regulation of Biotechnology, 51 Fed. Reg. 23,302 (June 26, 1986).

88 Presidential Commission, *supra* note 2.

89 Emily Waltz, *CRISPR-Edited Crops Free to Enter Market, Skip Regulation*, 34 Nat. Biotechnol. 582 (2016).

90
*Modernizing the Regulatory System for Biotechnology Products: Final Version of the 2017 Update to the Coordinated Framework for the Regulation of Biotechnology*, U.S. Food and Drug Admin. (2017), https://usbiotechnologyregulation.mrp.usda.gov/2017_coordinated_framework_update.pdf. See also Evans, *supra* note 83.

91
*Id*.

92 18 U.S.C. § 175 (2018) (biological weapons); 18 U.S.C. § 2332a (2018) (weapons of mass destruction).

93 E. Haavi Morreim, *Litigation in Clinical Research: Malpractice Doctrines Versus Research Realities*, 32 J. L. Med. & Ethics 474 (2004).

94 See Guerrini, *supra* note 82, at 1443.

95 Antonio Regalado, *Don’t Change Your DNA at Home, Says America’s First CRISPR Law*, MIT Technology Review (Aug. 9. 2019), https://www.technologyreview.com/2019/08/09/65433/dont-change-your-dna-at-home-says-americas-first-crispr-law/.

96 S.B. 180, 2019–2020 Reg. Sess. § 2 (Cal. 2019).

97 Regalado, *supra* note 95. Regalado states: ‘Asked for examples of products the bill could affect, Chang’s staff provided a link to an Amazon ad for a $159 box of supplies sold by Zayner, which “includes everything you need to make precision genome edits in bacteria at home.” Because that kit targets genes in bacteria, however, it would not have any effect in humans, and it is not clear why it would need a warning label’. *Id*.

98
*Screening Framework Guidance for Providers of Synthetic Double-Stranded DNA*, U.S. Dept. of Health and Human Services (2010), https://www.phe.gov/Preparedness/legal/guidance/syndna/Pages/default.aspx.

99 See *Risk-Based Performance Standards Guidance*, U.S. Dept. of Homeland Sec. (May 2009), https://www.dhs.gov/xlibrary/assets/chemsec_cfats_riskbased_performance_standards.pdf.

100 Zettler et al., *supra* note 34.

101
*Id*.

102 Nair, *supra* note 8, at 231 (emphasis added).

103 Landrain et al., *supra* note 2; *Draft DIYbio Code of Ethics from North American Congress*, DIYBio (July 2011), http://diybio.org/codes/code-of-ethics-north-america-congress-2011/ (The North American code consists of seven broad directives: ‘Promote citizen science and decentralized access to biotechnology; emphasize transparency, the sharing of ideas, knowledge and data; engage the public about biology, biotechnology and their possibilities; adopt safe practices; respect the environment; biotechnology should only be used for peaceful purposes; and tinkering with biology leads to insight [and] insight leads to innovation.’) [hereinafter North American Code].

104
*Draft DIYbio Code of Ethics from European Congress*, DIYBio (2011), https://diybio.org/codes/draft-diybio-code-of-ethics-from-european-congress/ [hereinafter European Code].

105 North American Code, *supra* note 103; European Code, *supra* note 104.

106 See Landrain et al., *supra* note 2, at 124; DIYBio, DIYBio Biosafety Advisory Service 2015 [Dec. 22, 2015]; DIY Bio Safety 2016 [Jan. 31, 2016].

107 Personal communication from Todd Kuiken (Nov. 16, 2020).

108
https://medium.com/p/dcc46e513036/edit.

109
*Community Ethics*, Global Community Bio Summit (2017), https://www.biosummit.org/ethics.

110 Jenny Molloy, *Launch of the Community Biology Biosafety Handbook*, Openbioeconomy (Oct. 11, 2020), https://openbioeconomy.org/blogpost/community-biology-biosafety-handbook/.

111
*Id*.

112
*Id*.

113
*Id*.

114
*Id*.

115
*Id*.

116
*Id*.

117 The Handbook notes that patient advocates as well as the patients themselves can contact NGE experimenters. *Id*.

118
*Id*.

119 See, e.g., *Join the Lab*, Genspace, https://www.genspace.org/join-the-lab (last visited July 25, 2022).

120
*Recognizing the Biosafety Levels*, Centers for Disease Control and Prevention, https://www.cdc.gov/training/quicklearns/biosafety/ (last visited July 25, 2022).

121
*About Us*, Counterculture Labs, https://www.counterculturelabs.org/info--history.html (last visited July 25, 2022).

122 Centers for Disease Control and Prevention, *supra* note 120.

123
*BioCurious Code of Conduct*, Google Docs., https://docs.google.com/document/d/e/2PACX-1vSUtixs69pJHSRkp05_Iy0nfWTDxwq9w6lFjU-n-bQYabTLHunp94Q5XDb36zoZQ6yEQ1JITG_5UmhE/pub (last visited July 25, 2022).

124 See, e.g., BioCurious Events, BioCurious, https://biocurious.org/events/ (last visited July 25, 2022).

125 Anna Wexler, Rebekah Choi, Alex Pearlman, & Lisa M. Rasmussen, *Navigating Biosafety Concerns within COVID-19 Do-it-Yourself (DIY) Science: An Ethnographic and Interview Study* (Aug. 16, 2022). Available at SSRN: https://papers.ssrn.com/sol3/papers.cfm?abstract_id=4191880.

126 Personal Communication from Daniel Vail. (Apr. 6, 2020).

127 DIYBio, *DIY Gene Therapy for Epigenome Reset*, Google Groups (May 13, 2020, 12:44 EDT), https://groups.google.com/g/diybio/c/2wdQZV2vQkI.

128
https://groups.google.com/g/diybio/c/5tXll5L83lA.

129 For details on laws and regulations, see *Laws and Regulations*, Nat’l Ass’n of Rocketry, https://www.nar.org/find-a-local-club/section-guidebook/laws-regulations/ (last visited July 25, 2022).

130 See *Drone Laws in the United States of America*, UAV Coach, https://uavcoach.com/drone-laws-in-united-states-of-america/ (last visited July 25, 2022).

131
*Id*.

132 See American Home Brewers Association, *Statutes*; Available at: https://www.homebrewersassociation.org/homebrewing-rights/statutes/united-states/.

133 See Guerrini et al., *supra* note 83, at 129–35.

134 Tim Collins, *Germany Cracks Down on BIOHACKERS: DIY Geneticists May Face Jail Time Following Fears They Could Create Bioweapons*, Daily Mail (Feb. 10, 2017), https://www.dailymail.co.uk/sciencetech/article-4211206/Biohackers-Germany-face-jail-time.html. The Group on Ethics in Science and New Technologies in the European Commission Directorate-General for Research and Innovation states that ‘German federal genome editing law, for example, provides that “genome editing can only be conducted in laboratories for genome editing” (free translation of the authors), (“Gentechnische Arbeiten dürfen nur in gentechnischen Anlagen durchgeführt werden”, Gesetz zur Regelung der Gentechnik’), *Opinion on Ethics of Genome Editing*, Eur. Comm’n (Mar. 2021), https://ec.europa.eu/info/sites/default/files/research_and_innovation/ege/ege_ethics_of_genome_editing-opinion_publication.pdf, at 33 n.61.

135 See Natalie Ram, *Science as Speech*, 102 Iowa L. Rev. 1187 (2017); Lori Andrews, *Is there a Right to Clone?* 11 Harv. J. L. & Tech. 643 (1998).

136 See *supra* text accompanying notes 96 and 97.

137 New Jersey at one point attempted to impose a licensing requirement on home brewing but backed off after lobbying opposition. See *supra* note 128.

138 See 47 C.F.R. §§97.1–97.527 and Appendix 2 (2022). For the history of ham radio regulation, see Jim Maxwell, *Amateur Radio: 100 Years of Discovery*, QST (Jan. 2000), http://www.arrl.org/files/file/About%20ARRL/Ham_Radio_100_Years.pdf (As Maxwell states, ham radio emerged in the early 20th century. Potential public benefits such as facilitating emergency communication during natural disasters were identified, but also concerns about interference between amateurs and government or commercial channels. In response, Congress passed the Radio Act of 1912, which required amateur operators to be licensed and restricted them to a single wavelength of 200 m. This caused a drop-off in ham radio use, leading operators in 1914 to organize the American Radio Relay League (ARRL) to represent and lobby for their interests. The value of ham radio was recognized during the First World War, but the practice was conducted under military supervision. The Navy wanted to continue to control the practice after the war, but the ARRL opposed this and independent amateur radio returned. In 1927, the federal Radio Act gave authority over amateurs to the Federal Communications Commission. In the 1950s, the FCC created different classes of amateur licenses, but there were few differences between what the classes could do, so in the 1960s, the FCC adopted ‘incentive licensing’, which gave higher classes greater access to wavelengths. Localities imposed restrictions and zoning rules to control the location of ham radio towers, which led to litigation. The FCC has issued a preemptive ruling stating that local governments must reasonably accommodate amateur operations, but they may still zone for height, safety, and aesthetics concerns. (Federal preemption of state and Local Regulations Pertaining to Amateur Radio Facilities, 101 F.C.C. 2d 952 [1985]; Pentel v. City of Mendota Heights, 13 F.3d 1261 [8th Cir. 1994]). In addition to being licensed by the FCC, operators must notify the commission if they propose to erect tall antennas near airports and must apply for call signs. Violations of FCC requirements come with criminal penalties.).

139 Landrain et al., *supra* note 2.

140 Caplan et al., *supra* note 58.

141 See Wexler et al., *supra* note 125.

142 Presidential Commission, *supra* note 2.

143 For example, there is no crime of auto-mayhem.

144 See *supra* text accompanying notes 99–104.

145 See DIYBio, https://diybio.org/ (last visited July 25, 2022).

146
*Id*.

147 See *supra* text accompanying notes 105–9.

148
*Id*.

149 Steve Mann et al., *Code of Ethics on Human Augmentation,*Wearcam (June 2016), http://wearcam.org/code.pdf.

150 See *supra* note 126.

151 See *Safety Codes*, Nat’l Ass’n of Rocketry, https://www.nar.org/safety-codes-2/ (last visited July 25, 2022); *Tripoli Rocketry Association Safety Code*, Tripoli Rocketry Ass’n (effective May 1, 2022), https://www.tripoli.org/content.aspx?page_id=22&club_id=795696&module_id=520420.

152 Laws and Regulations, *supra* note 129.

153 For a history of the role of amateur rocket clubs in rocketry, see Oliver Morton, *The Moon* (2019).

154 See Acad. of Model Aeronautics, https://www.modelaircraft.org/ (last visited July 25, 2022), Drone User Group Network, Crunchbase, https://www.crunchbase.com/organization/drone-user-group-network (last visited July 25, 2022).

155 See Am. Homebrewers Ass’n, https://www.homebrewersassociation.org/ (last visited July 25, 2022).

156 See *supra* text accompanying notes 115–39.

157 See *Safety and Security Hub*, iGEM (2021), https://2021.igem.org/Safety.

158 See *supra* text accompanying note 81.

159 Meredith Trejo et al., *‘A Cohort of Pirate Ships’: Biomedical Citizen Scientists’ Attitudes Toward Ethical Oversight*, 6 CSTP 15 (2021).

160 Jessica Richman & Zachary Apte, *Crowdfunding and IRBs: The Case of uBiome*, Sci. Am. Blog Network (July 22, 2013), https://blogs.scientificamerican.com/guest-blog/crowdfunding-and-irbs-the-case-of-ubiome/.

161
*Id*. In 2021, Richman and Apte were indicated by a federal grand jury and charged with multiple federal crimes including ‘conspiracy to commit securities fraud, conspiracy to commit health care fraud, money laundering, and related offenses in connection with alleged schemes to defraud health insurance providers and investors to raise capital for now-bankrupt microbiome testing company uBiome’. They fled the country and are now fugitives. uBiome Co-Founders Charged With Federal Securities, Health Care Fraud Conspiracies, DOJ (Mar. 18, 2021), https://www.justice.gov/usao-ndca/pr/ubiome-co-founders-charged-federal-securities-health-care-fraud-conspiracies.

162 See *Collaborative Norm Setting Framework*, Alex Pearlman, https://www.alexpearlman.com/norms (last visited July 25, 2022).

163 See JOGL Fitz, https://app.jogl.io/space/joglfitz (last visited July 25, 2022).

164 See Jason Bobe, *Ask a Biosafety Expert: User-Driven Advisory Service for the Do-It-Yourself Biology (DIYBio) Community*, Slideshare (Oct. 23, 2013), https://www.slideshare.net/JasonBobe/ask-a-biosafety-expertabe-10232013. For a description of these credentials, see *Professional Credentials in Biosafety*, Ass’n for Biosafety and Biosecurity, https://absa.org/credentials/ (last visited July 25, 2022).

165 Funding from foundations, crowdfunding or the government could subsidize liability insurance premiums. The premiums for the members of the Ask a Safety Expert Service were approximately $2500 per year. The challenge of securing these funds was the main reason that the service was discontinued. Personal Communication from Todd Kuiken, Dec. 16, 2020.

166 See Richman and Apte, *supra* note 161.

167 See *Insurance Questions*, Nat’l Ass’n of Rocketry, https://www.nar.org/safety-information/insurance-questions/ (last visited July 25, 2022).

168 See *Special Risk Insurance Certificate Issued by Federal Insurance Company for Academy of Model Aeronautics*, Modelaircraft (effective Jan. 1, 2014) https://www.modelaircraft.org/sites/default/files/500-m.pdf.

169 See *Homebrew Club Insurance*, Am. Homebrewers Ass’n (2022), https://www.homebrewersassociation.org/homebrew-clubs/homebrew-club-insurance/.

170 See Jonathan Kahn, *Synthetic Hype: A Skeptical View of the Promise of Synthetic Biology*, 45 Val. U. L. Rev. 1343, 1359 (2011) (‘Institutional Biosafety Committees (“IBCs”) … were established under the NIH Guidelines for Recombinant DNA Research “to assess the biosafety and environmental risks of proposed recombinant DNA experiments conducted in academic and commercial settings, and to decide on the appropriate level of biocontainment.” While established under federal guidelines, such bodies are fundamentally private in nature and ultimately a form of self-regulation by the institutions that maintain them’.

171 See *supra* text accompanying notes 121–3.

172 Molloy, *supra* note 110.

173
*Id*.

174 See *supra* text accompanying note 60.

175 See *supra* text accompanying note 110*.*

176 For example, Texas makes it a misdemeanor to fail to report a felony that causes serious bodily harm or death, but only when someone actually observes the felonious activity. See Texas Penal Code §38.171.

